# A complex adaptive systems model of labour reciprocity and normative reasoning in swidden agriculture

**DOI:** 10.1098/rsos.242197

**Published:** 2025-05-21

**Authors:** Denis Tverskoi, Shane A. Scaggs, Sean S. Downey

**Affiliations:** ^1^ Health and Environment Modeling Lab, The Ohio State University, Columbus, OH, USA; ^2^ Division of Biostatistics, College of Public Health, The Ohio State University, Columbus, OH, USA; ^3^ Department of Anthropology, The Ohio State University, Columbus, OH, USA; ^4^ The Sustainability Institute, The Ohio State University, Columbus, OH, USA; ^5^ The Translational Data Analytics Institute, The Ohio State University, Columbus, OH, USA

**Keywords:** swidden agriculture, Indigenous societies, complex adaptive systems, sustainability science, mathematical modelling, cultural evolution

## Abstract

Swidden (aka ‘slash-and-burn’) agriculture is a prototypical-coupled human and natural system. Despite this, mathematical models integrating its social and ecological dynamics are rare. Here, we use complex adaptive systems theory to develop a model where individuals rely on labour exchange driven by reciprocity and normative reasoning that can lead to sanctions. Our results identify three emergent regimes: low-intensity swidden, sustainable high-intensity swidden that maximizes ecosystem services and harvest returns, and deforestation. We show that sustainable high-intensity swidden evolves if labour reciprocity and normative reasoning are balanced: helping behaviour should be significantly conditioned by normative reasoning to prevent over-harvesting, while reciprocity is necessary to prevent excessive sanctioning. We find that the sustainable high-intensity swidden regime is robust to changes in group size, is resilient to environmental shocks, can evolve under various models of forest ecology and is most productive for both forests and farmers when the balance of labour reciprocity and normative reasoning results in an intermediate scale of forest disturbance. Overall, we illuminate the importance of Indigenous social norms and customary practices related to swidden labour in maintaining sustainable and intensive swidden agriculture.

## 1. Introduction

Swidden involves the intentional use of fire to clear primary or secondary forests to create agricultural fields [1]. Fire eliminates pests and releases organic matter and nutrients bound in plant biomass into the topsoil. In the swidden farming cycle, short-term cultivation is typically followed by relatively long periods of fallowing [1,2]. This type of farming has been practised for thousands of years [3–5] and is now widely used in Indigenous and smallholder communities in tropical and subtropical regions, including Central and South America, South Asia and Africa [6,7]. Estimates suggest that between 300 million [8] and 1 billion people [5] rely on swidden agriculture to some extent. In 2018, Indigenous peoples controlled 37% of the natural land on the Earth [9]. About 36% of intact forests [10], 60% of all well-known terrestrial mammals [11] and 24% of the total above-ground carbon in the world’s tropical forests [12] are on Indigenous peoples’ lands. These estimates indicate that swidden agriculture, along with other traditional forms of forest-based subsistence, significantly affect global climate dynamics and sustainability, as Pisor *et al*. demonstrated in a recent special issue, ‘Climate change adaptation needs a science of culture’ [13]. Therefore, understanding and acknowledging the role of Indigenous swidden practices in biodiversity conservation and forest stewardship are crucial [14].

The literature on swidden is vast and has been reviewed elsewhere (e.g. [15]); however, we provide a brief overview here. Historically, perspectives on the effects of swidden agriculture on the environment and the conservation of tropical forest ecosystems have been polarized. Some research emphasizes its negative effects on the environment, hypothesizing that it typically leads to soil degradation and deforestation—especially in the context of population growth—and advocates for the ‘modernization’ of traditional agricultural practices (see [5] for examples). A substantial amount of research relates to swidden farming practices such as the length of the fallow period, managing crop diversity, land preparation methods and traditional ecological knowledge [16–19]. An increasing number of researchers are also using an integrated socioecological system (SES) approach in which human social, cultural and demographic factors coevolve with tropical forest ecosystem dynamics [8,20–22]. This approach typically views swidden agriculture as a productive and sustainable system that can under some circumstances enhance the forest biodiversity [19,23–26]. A smaller body of research examines how social factors such as social structure, social norms, religion and rituals may also be important determinants of sustainable swidden cultivation [20,27]. Indeed, swidden farming typically involves numerous labour-intensive tasks such as forest clearing, burning, planting, weeding and harvesting, and many of these activities can be performed more safely and efficiently when people work together in groups. As a result, both social and environmental factors contribute to the dynamics of labour exchange networks and may also shape the trajectory and outcome of local cultivation practices. Clearly, a deep understanding of both the social and environmental drivers of sustainable swidden agriculture is crucial for the conservation of tropical ecosystems and the mitigation of climate change.

Long-term ethnographic research in Maya communities that use swidden cultivation in Southern Belize indicates that community forests are a common resource, with no formal institution or leader regulating individual cultivation practices [27]. Consequently, swidden labour exchange networks result from social norms related to cooperation rather than institutional leadership. One such norm is direct reciprocity [28]. Direct reciprocity is defined as ‘a mechanism where people help those who have helped them in the past’ [29]. A household survey conducted in two Maya villages in 2018 revealed that direct reciprocity is a significant determinant of the structure of the labour exchange networks associated with swidden cultivation, but that there are also asymmetric labour exchange patterns [30]. One possible explanation for this asymmetry is what we define here as *normative reasoning and sanctioning* (or simply, *normative reasoning*)—the cognitive process by which individuals evaluate whether to provide swidden labour to a partner requesting help, based on perceptions, beliefs or understanding of the acceptability of the request. This decision process was documented in an experimental study involving 150 participants from the same two villages [27]. In the experiment, participants played a common pool resource (CPR) game that simulated swidden cultivation practices in the village. The environment was represented as a grid in which each cell was either occupied by trees or empty, and the rate of forest regeneration increased with the number of forest cells remaining on the grid. Groups of five players simultaneously chose the number of cells they would like to clear, and the game was played over several rounds. In the first experiment, no helpers were required to clear new fields so the behaviour of participants in almost all groups converged close to the Nash equilibrium, indicating that participants cleared the maximum possible number of cells in each round, which in turn led to rapid deforestation. In the second experiment, each player was able to clear one cell on their own, but clearing more cells required the help of others, simulating labour exchange needs in the villages. The results demonstrate a significant increase in the remaining fraction of forest among groups. We suspect that this is because participants used normative reasoning when making their helping decisions: information about each player’s requests were displayed to the group and the results suggested that they used this information to decide whether to provide help, depending on how acceptable they thought the request was. In this way, players could effectively ‘sanction’ [31] a player who wanted to clear relatively large fields by refusing to help them, and they could help those who requested smaller fields that they thought were less likely to threaten the common-pool forest resource. Support for this sanctioning mechanism was noted in post-game interviews by one participant: ‘... some ask for five. If they ask for five, they shouldn’t be helped but if you ask for three, that is a good piece of land and it considers other people’ (quoted in [27])^1^. Here we build on these experimental and ethnographic results by employing mathematical modelling to better understand theoretically how normative reasoning and labour reciprocity may shape the evolution of sustainable swidden agriculture.

The existing modelling literature on CPR games is extremely large [31,32]. Here, we focus on dynamical CPR games with explicit resource dynamics. There are two approaches to modelling such situations. The first approach employs classical game theory assuming that all agents are perfectly rational, so they have the correct beliefs about the behaviour of others and the resource dynamics [33–36]. Such agents are postulated to maximize their expected discounted utility (pay-off) over all future rounds [33,36]. Although this approach is analytically tractable, the assumption of perfect rationality is highly questionable as it implies that agents are able to correctly predict the behaviour of others, the state of the environment and choose the best action given these predictions (for a more detailed discussion, see [37]), often ignoring the effects of cognitive, cultural and social factors [38]. An alternative approach assumes bounded rationality, meaning that agents do not fully predict the behaviour of others or the dynamics of the environment. Evolutionary game theory provides a simple, elegant and effective framework for modelling the behaviour and evolution of bounded rational agents. Typically, three types of agents are considered in models employing evolutionary game theory: punishers, cooperators and defector. Cooperators and punishers are prescribed to extract fewer resources than defectors. Punishers sanction defectors, imposing costs on defectors but also being costly for punishers [34,39,40]. The within-group dynamics that determine how the frequencies of the three types of agents change are governed by the diffusion of harvesting strategies that generate above-average pay-offs, resulting in replicator dynamics. Potential mechanisms leading to such dynamics can be natural selection (genetic evolution), where maladapted agents ‘die’ and are removed from a simulation, and payoff-based imitation (cultural evolution), where maladapted agents adopt strategies from agents with higher pay-offs. Here, we build a parsimonious agent-based model using a minimalist cultural evolutionary framework. We account for the individual decision-making process by assuming bounded rationality of agents, and we model the social dynamics of swidden labour and a range of simple forest growth dynamics. As a result, we contribute to the literature on modelling dynamical CPR games by considering punishment in the context of labour exchange networks, which is relevant in many natural resource management contexts.

Many existing models of swidden agriculture examine land use and land cover change (LULCC) patterns by linking raster images from remote sensing data with agent-based models of human land use [41–45]. Typically, these studies present relationships between demographic variables such as household size, village population levels and land use rates with different levels of agricultural or economic efficiency and model the decision-making process of agents using assumptions of perfect or bounded rationality [46]. While many existing LULCC swidden models include computationally sophisticated, theoretically informed mechanisms for predicting patterns of land use change in space and time, or the effects of nature conservation policy [45], they typically do not incorporate social or cultural factors that can affect land use. A second category of models examines swidden as an integrated SES with dynamic, integrated social and environmental modelling elements [47–50]. Most of these models have a large number of free parameters or incorporate raster images from real study locations and therefore they cannot be considered parsimonious models. Nor do they typically account for important social, cultural and behavioural determinants of swidden agriculture from local communities, with the exception of the demography. In this respect, the model developed by Barton [21] stands out for its parsimonious design and because it examines settlement patterns related to swidden agriculture and the underlying household, social and environmental dynamics, including social norms related to land ownership in the ancient American Desert Southwest.

Barton’s model is particularly relevant to our current study because it applies complex adaptive systems (CAS) theory to agent-based modelling and archaeology. CAS theory proposes that most complex systems are characterized by nonlinearity and sensitivity to initial conditions, and that low-level interactions among agents can lead to emergent system-level properties [51]. Previously, Downey *et al*. [25] argued that CAS theory is a useful heuristic model for swidden agriculture. The mathematical model we present here follows up on that suggestion. It differs from most of the swidden models reviewed above: it is a parsimonious model that is designed for a comprehensive theoretical exploration of the coupled dynamics of social and behavioural determinants of swidden agriculture. It accounts for social norms related to swidden agriculture, a range of simple ecological models and emergent properties related to the long-term sustainability of the coupled human and natural system.

Our review of work in evolutionary theory, modelling of CPR games and computational models of swidden agriculture reveals a gap in our theoretical understanding of the relationship between social norms and customary practices in swidden commons. Here, we hypothesize that labour reciprocity and normative reasoning based on individual perceptions of acceptable land use rates will determine the trajectory and the outcome of the coupled socioecological swidden system. However, it remains unclear (i) how individual perception of acceptability coevolves with the ecosystem and relates to sustainability; (ii) how the interplay of reciprocity and normative reasoning shapes the dynamics of this SES; and (iii) what are the joint effects of the above socio-behavioural factors with material, demographic and ecological factors on the dynamics and sustainability of swidden cultivation. Addressing these issues theoretically is the goal of this article. To do this, we use a CAS framework to develop a mathematical model that focuses on the labour exchange dynamics taking place in groups practising swidden cultivation in community forests. In doing so, we hope to make the following contributions: first, we contribute to the literature on common pool resource games by modelling the effects of social sanctioning when labour exchange networks are coupled with simple ecological models. Second, we illuminate the importance of social norms and customary practices related to swidden labour in maintaining sustainable and intensive swidden agriculture in community forests worldwide. Third, we document our model in a reproducible manner so that other researchers can adapt and integrate it in future research and policy applications.

## 2. Methods

We consider a group of N agents interacting on an L×L grid over T rounds. We treat this group as a village and each agent as a household.

### 2.1. Ecological dynamics

Following [52], we postulate each cell k is either occupied by trees or empty. Note that we intentionally simplify the ecological dynamics and do not model the different stages of forest succession because our focus is on the social dynamics shaping the evolution of sustainable swidden agriculture (see [25] for an analysis of succession in swidden forests). We assume that each empty cell turns into forest with probability $p(f_{k})$, which depends on the fraction $f_{k}\in [0,1]$ of neighbouring cells^2^ occupied by trees. We postulate that this probability peaks at fraction F. For simplicity, we assume that $p(f_{k})$ grows linearly from $p_{0}$ to $p_{max}$ as $f_{k}$ increases from 0 to F and decreases linearly from $p_{max}$ to $p_{1}$ as $f_{k}$ increases from F to 1


(2.1) $$p(f_{k})=\left\{ \begin{array}{l} (p_{max}-p_{0})\frac{f_{k}}{F}+p_{0},\text{ if }f_{k}\leq F,\\ \frac{1}{1-F}[p_{max}-p_{1}F-(p_{max}-p_{1})f_{k}],\text{ otherwise}. \end{array}\right.$$

With F≈0.5, the probability that an empty cell will regenerate into forest peaks at moderate levels of local forest density, while with F close to 1, the above probability peaks at maximum local forest density (i.e. when the neighbourhood surrounding a disturbed cell is entirely composed of forest). The latter case assumes that the regeneration of a disturbed cell increases with local forest density and can be approximately described by logistic growth (for more details see electronic supplementary material, section S2.2). In contrast, the former case relaxes this assumption, reflecting a more substantial dependence of germination on competition for resources [53]. We define the amount of new biomass $\Delta_{f}^{+}$ as the number of empty cells regenerated into forest in the current round, and the fraction of cells f occupied by trees at the end of the round. Varying parameters $\left(F,p_{0},p_{1},p_{max}\right)$ allow us to examine the effects of different environmental conditions on the model dynamics.

### 2.2. Agents and their actions

We posit that each agent i is characterized by their clearing request $x_{i}\geq 0$ and their sanctioning (acceptability) threshold $y_{i}\geq 0$. The clearing request $x_{i}$ is the number of cells i wants to clear given the actual number of cells occupied by trees. Following the standard convention in the common-pool resource literature, we assume that each agent can clear at most N-th fraction of the total number of cells occupied by trees [36]. However, to clear these cells, i needs help from others [27].

### 2.3. Dynamics of helping behaviour

We assume two factors contribute to the propensity of agent i to help j with the request $x_{j}$: normative reasoning and reciprocity. First, i tends to reject requests they perceive unacceptable (i.e. those with $x_{j}>y_{i}$) and provide help in response to acceptable requests (i.e. those with $x_{j}\leq y_{i}$). Second, helping decisions are also driven by direct reciprocity: agents tend to help more group-mates who have helped them in the past, where the history of help received by i from j is captured by value $h_{j,i}\in \left[0,1\right]$. The parameter μ∈[0,1] captures the relative importance of normative reasoning in making helping decisions, with respect to the expectation for reciprocity given the helping history of $h_{j,i}$. As a result, the probability $q_{i,j}$ that i will help j (j≠i)^3^ with the request $x_{j}$ is


(2.2) $$q_{i,j}=\underset{\text{reciprocity}}{\underbrace{(1-\mu )h_{j,i}}}+\underset{\text{normative reasoning}}{\underbrace{\mu I_{\left\{x_{j}\leq y_{i}\right\}}}},$$

where $I_{\left\{x_{j}\leq y_{i}\right\}}$ is an indicator function equal to 1 if the clearing request $x_{j}$ does not exceed the threshold $y_{i}$, and 0 otherwise. Equation (2.2) implies that with μ close to zero, helping behaviour is driven primarily by reciprocity. As μ increases, helping behaviour becomes increasingly conditioned by normative reasoning. Overall, one can treat $y_{i}$ as personal sanctioning norm, and $\bar{y}$ as a social sanctioning norm. Given a fixed relative importance of normative reasoning μ, relatively low values of $\bar{y}$ correspond to more severe sanctions, while relatively high values of $\bar{y}$ correspond to milder sanctions.

Finally, the history of labour exchange interactions is updated based on the current history and current helping decisions with weights (1−α) and α∈[0,1]*,* respectively:


(2.3) $$\begin{array}{r} h_{i,j}^{\prime }=\left\{ \begin{array}{l} (1-\alpha )h_{i,j}+\alpha ,\text{if }i\text{ helped }j\text{ in the current round},\\ (1-\alpha )h_{i,j},\text{otherwise}, \end{array}\right. \end{array}$$

where the prime denotes the next round.

### 2.4. Individual harvest and pay-offs

The individual harvest $x_{a,i}$ (i.e. the number of cells cleared by agent i) is determined by the clearing request $x_{i}$ and the number of helpers $N_{i}^{+}$. Following [27], we posit that the number of cells i is able to clear has a linear relationship with the number of helpers. Specifically, an agent alone (i.e. when $N_{i}^{+}=0$) is able to clear h cells. Each helper allows an additional h cells to be cleared. The individual pay-off $\pi_{i}$ is determined by the individual harvest $x_{a,i}$ and total costs $C_{i}$


(2.4) $$\pi_{i}=\underset{\text{harvest, }x_{a,i}}{\underbrace{min\{x_{i},h(1+N_{i}^{+})\}}}-\underset{\text{total costs}}{\underbrace{C_{i}}}.$$

We postulate there are two types of costs in the model. A fixed cost $c_{0}\geq 0$ is imposed on agent i≠j helping another agent j and is independent of the number of helpers of j. This cost can be treated as the transportation cost or as psychological discomfort from doing something outside of the normal routine. We also assume that each helper of j (including j) incurs the maximum cost c≥0 if they clear h cells, and this cost is proportionally reduced if fewer than h cells are cleared (this depends on the number of helpers $N_{j}^{+}$). As a result, by helping j, agent i incurs costs


(2.5) $$c_{i,j}=\underset{\text{fixed costs}}{\underbrace{c_{0}I_{\left\{i\neq j\right\}}}}+\underset{\text{variable costs}}{\underbrace{\frac{x_{a,j}}{h(1+N_{j}^{+})}\cdot c}},$$

where $I_{\left\{i\neq j\right\}}$ is an indicator function equal to 1 if i≠j and 0 otherwise. Then the total costs $C_{i}$ are the sum of the costs $c_{i,j}$ over all agents j who received help from i.

### 2.5. Strategy revision protocol

We assume that each round, each agent i is independently given an opportunity to revise their clearing request $x_{i}$ (using myopic optimization) and sanctioning threshold $y_{i}$ (using pay-off-based imitation and innovations) with probabilities $\nu_{0}\in \left[0,1\right]$ and $\nu_{1}$+$\nu_{2}\in \left[0,1\right]$*,* respectively. Myopic optimization implies an agent chooses a strategy maximizing their pay-off while keeping all others’ decisions the same as in the previous round [54–56]. Myopic optimization reflects bounded rationality of agents who are able to maximize their pay-off but unable to predict changes in the behaviour of others. Here we implement myopic optimization as follows. Agent i with the clearing request $x_{i}$ forms a belief on the number of helpers in the current round based on its expected value given the request $x_{i}$, the current history of labour exchange interactions and the previous sanctioning thresholds of others, i.e. ${\tilde{N}}_{i}^{+}(x_{i})=E(N_{i}^{+})(x_{i})$. Agent i then chooses the clearing request $x_{i}$ maximizing their material pay-off $\pi_{i}(x_{i},{\tilde{N}}_{i}^{+}(x_{i}))$ given the expectations on the number of helpers (for more details see electronic supplementary material, section S2.2).

Since individual decisions on sanctioning thresholds affect not the current but next round pay-offs and are also conditioned by the choice of clearing requests in the next round, using myopic optimization to revise sanctioning thresholds seems cognitively very difficult—meaning that an agent should make predictions not only about the effects of their current choice on the number of helpers in the next round but also about their potential clearing request in the next round. To avoid this complexity, we assume agents update their threshold decisions using pay-off-based imitation with random innovations [21,57–60] with probabilities $\nu_{1}$ and $\nu_{2}$, respectively, ($\nu_{1},\nu_{2}\geq 0$). To implement errors in the imitation process, we use a quantal response equilibrium-like approach [61] with logit errors and a non-negative precision parameter λ. Namely, we posit that agent i copies the threshold $y_{j}$ of agent j with probability proportional to the *j*’s pay-off $\pi_{j}$


(2.6) p(yi′=yj)=eλπjNeλπ.

With λ→∞, i copies exactly the agent that has the highest pay-off, while with λ=0, i chooses who to copy completely randomly.

**Figure 1.**
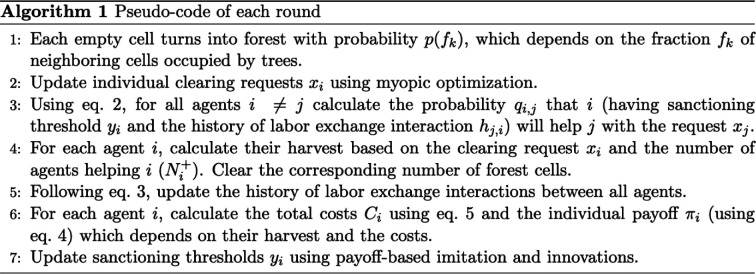
Pseudo-code of each round of the model.

Overall, the effect of the sanctioning (acceptability) threshold $y_{i}$ on the model dynamics is not trivial. Increasing $y_{i}$ implies i treats more clearing requests as acceptable, which in turn motivates i to help others more. On the one hand, this increases costs associated with helping others. On the other hand, it leads to more help received from others in the next round due to reciprocity but may undermine the ecosystem sustainability. As a result, the interplay of normative reasoning, reciprocity and costs of helping may produce non-trivial effects on the model dynamics. Our goal here is to understand how these factors, along with demographic and environmental parameters, shape the evolution of swidden agriculture. The main model parameters and outcomes are summarized in table 1. The primary algorithm used in the model is outlined in figure 1. The model description in the overview, design concepts and details format is presented in electronic supplementary material, section S1.

**Table 1.** Main parameters and outcomes of the model.

parameters	their meaning
μ	importance of normative reasoning
N	number of agents
$c_{0}$ and c	cost parameters
F	environmental parameter

outcomes	
$\bar{x}$	average clearing request
${\bar{x}}_{a}$	average harvest
$\bar{y}$	average sanctioning threshold
${\bar{N}}^{+}$	average number of helpers
f	remaining fraction of forest
$\Delta_{f}^{+}$	new biomass
$\bar{\pi }$	average pay-off

## 3. Results

Some analytical progress is possible for special cases of the model (electronic supplementary material, sections S2.2–S2.4), but to study the overall model dynamics, we employ agent-based simulations. We use the following set of default parameter values: N=40, L=120, $T=10^{3}$, $p_{0}=0.01$, $p_{1}=0.06$, $p_{max}=0.12$, F=0.5, h=3, $c_{0}=0.1$, c=0.2, α=0.3, μ=0.75, λ=10, $\nu_{0}=\nu_{1}=0.2$ and $\nu_{2}=0.1$. Initial clearing requests $x_{i}$ are drawn randomly and independently from a discrete uniform distribution on {1,..,5}*,* while initial sanctioning (acceptability) thresholds $y_{i}$ are drawn randomly and independently from a uniform distribution on {0,..,hN}. We posit that initially all cells are occupied by trees (i.e. $f_{0}=1$). All initial helping histories $h_{i,j}$ are set to zero. These conditions suggest that initially there is no consensus on what size clearing request (y) is acceptable, so it can vary from a minimum value of $y_{i}=0$, which indicates agent i considers clearing any amount of forest to be completely unacceptable, to a maximum value which is limited by the group size $y_{i}=hN$, which means agent i considers any clearing request to be acceptable. We define outcomes as *sustainable* if the average remaining fraction of forest $\bar{f}$ in the last 25% of rounds is greater than 0.05 and *unsustainable* otherwise.

We vary parameters F, μ, N, $c_{0}$ and c to examine under what conditions sustainable swidden agriculture can evolve. In these settings, three types of swidden regimes are identified.

### 3.1. Three swidden regimes

**Figure 2.**
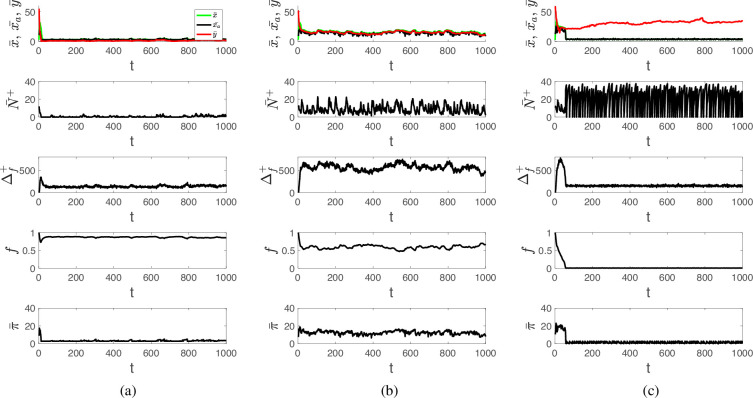
Examples of three swidden regimes: (a) low-intensity swidden (the relative importance of normative reasoning μ=1), (b) sustainable high-intensity swidden (μ=0.75) and (c) deforestation (μ=0.75). Shown are the dynamics of the average clearing request $\bar{x}$, the average harvest ${\bar{x}}_{a}$, the average sanctioning threshold $\bar{y}$, the average number of helpers ${\bar{N}}^{+}$, the new biomass $\Delta_{f}^{+}$, the remaining fraction of forest f and the average pay-off $\bar{\pi }$. Parameters are at default values, except as indicated.

The low-intensity swidden regime is characterized by very severe sanctions (i.e. $\bar{y}$ is closed to 0) resulting in no helping behaviour (figure 2a), so that each agent clears fields individually, and a total of approximately hN cells are cleared in each round. As a result, the remaining fraction of forest is very high (for the derivation of this equation see electronic supplementary material, section S2.2)


(3.1) $$f^{*}=\frac{1}{2}+\frac{p_{max}-p_{1}F}{p_{max}-p_{1}}-\frac{1-F}{p_{max}-p_{1}}\sqrt{p_{1}^{2}+4\nu h\frac{p_{max}-p_{1}}{1-F}},$$

where ν is the population density. Clearly, the outcome is characterized by forest conservation but is inefficient in terms of the agents’ economic productivity, and harvest returns (${\bar{x}}_{a}$) may be insufficient for household needs.

**Figure 3.**
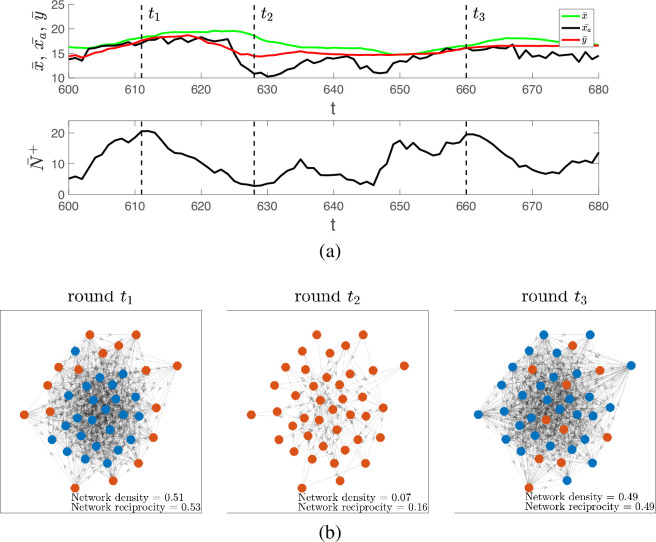
Visualization of cycles of the sanctioning norm $\bar{y}$, harvest ${\bar{x}}_{a}$ and helping behaviour ${\bar{N}}^{+}$. (a) Zoomed-in dynamics of the sanctioning norm, harvest and helping behaviour shown in figure 2b. (b) The corresponding dynamics of the labour exchange network in three rounds: $t_{1}$ (relatively loose sanctioning norm $\bar{y}$ and high compliance with it resulting in a dense labour exchange network), $t_{2}$ (relatively tight norm $\bar{y}$ and no compliance with it leading to a sparse labour exchange network driven solely by reciprocity) and $t_{3}$ (end of the cycle, similar to $t_{1}$). Nodes represent agents and edges correspond to labour exchange interactions between agents. Agents with relatively acceptable clearing requests (i.e. those with $x_{i}\leq \bar{y}$) are coloured blue, while agents with relatively unacceptable requests (i.e. those with $x_{i}>\bar{y}$) are coloured red.

The sustainable high-intensity swidden regime is characterized by cycles of the sanctioning norm $\bar{y}$, the average harvest ${\bar{x}}_{a}$, helping behaviour ${\bar{N}}^{+}$ and the remaining fraction of forest f (figure 2b). Cycling occurs because increasing the norm $\bar{y}$ weakens sanctions and promotes helping behaviour, which is further reinforced by labour reciprocity when its importance (1−μ) is significantly high. This process is accompanied by a reduction in the remaining fraction of forest, f. As the labour exchange network becomes significantly denser, further increases in the threshold y are disadvantageous because the associated costs of helping c outweigh the potential for increased harvests that results from having more helpers (figure 3, $t_{1}$)^4^. Moreover, it becomes beneficial for agents to increase their clearing requests x. This leads to higher harvests due to a dense helping network, and the breakdown of labour exchange connections if the relative importance of normative reasoning μ is sufficiently high, which ultimately decreases harvest and limits forest exploitation. In the state with a relatively sparse labour exchange network and relatively severe sanctions (figure 3, $t_{2}$), agents are motivated to reduce their clearing request x and/or increase their personal norm y to receive more help; however, this only works if costs of helping and the relative importance of normative reasoning μ are not very high. This tendency leads to an increase in $\bar{y}$ closing the cycle of norms, harvest, helping behaviour and forest dynamics (figure 3, $t_{3}$). Note that the fluctuations in $\bar{y}$ exhibit less variability than the fluctuations in ${\bar{N}}^{+}$ and $x_{a}$.

The deforestation regime occurs when f≈0 (see figure 2c). It is characterized by high levels of labour reciprocity and very mild sanctions (i.e. $\bar{y}$ is high), which cause rapid and widespread forest loss.

Next, we analyse the effects of social, demographic, material and environmental factors on the model dynamics. To ensure the robustness of our results, we perform 100 runs for each parameter combination. For further details on how the number of simulation runs affects result robustness, see electronic supplementary material, section S3.

### 3.2. Effects of social and demographic factors

**Figure 4.**
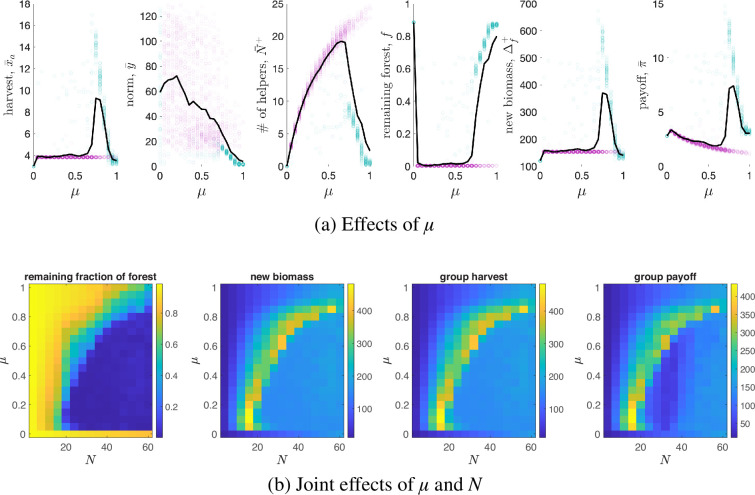
(a) Effects of the relative importance of normative reasoning μ on the main model outcomes. Shown are averages in different runs (points) and across all runs (solid black lines). Violet points correspond to unsustainable runs (i.e. those with f≤0.05), while green points correspond to sustainable runs (i.e. those with f>0.05). For each parameter combination, we performed 100 independent simulation runs and in each run, the simulation consists of 1000 rounds. Results in each run are averages based on the last 250 rounds. All parameters are at default values. (b) Joint effects of μ and the group size N on: the remaining fraction of forest f, the new biomass $\Delta_{f}^{+}$, the group harvest $x_{a}$ and the group pay-off π. Shown are the averages based on 100 runs each of 1000 rounds for each parameter combination. Results in each run are averages based on the last 250 rounds. Parameters are at default values.

First, we report the effects of the relative importance of normative reasoning μ on the model dynamics and outcomes (figure 4a). When the relative importance of normative reasoning μ is low but non-zero, helping behaviour is mainly driven by reciprocity, and the system approaches a unique equilibrium characterized by complete deforestation that is caused by very intense helping behaviour. Increasing μ leads to bifurcation and bi-stability with the co-existence of two stable equilibria: complete deforestation and sustainable high-intensity swidden agriculture. Around the bifurcation point, sustainable outcomes are characterized by the mildest sanctions and hence the highest harvest and pay-offs. As μ increases, helping behaviour becomes increasingly conditioned by normative reasoning and the frequency of sustainable outcomes increases, but harsher sanctions also evolve, leading to lower harvests and pay-offs. As μ approaches 1, the sustainable high-intensity swidden regime transforms into a low-intensity swidden regime characterized by no helping behaviour, small harvests, strong sanctions and relatively undisturbed forests. As a result, the sanctioning norm $\bar{y}$ decreases with μ, and the remaining fraction of forest f exhibits an S-shaped dependence on μ^5^. However, the average harvest ${\bar{x}}_{a}$, the average number of helpers ${\bar{N}}^{+}$, the new biomass $\Delta_{f}^{+}$ and the average pay-off $\bar{\pi }$ exhibit hump-shaped dependencies on μ. This implies the most socially and environmentally efficient outcomes with respect to harvests and biomass are observed at intermediate values of μ around the bifurcation point. Overall, the main takeaway is that the balance between normative reasoning and reciprocity is crucial for the evolution of sustainable and intensive swidden agriculture. Indeed, labour exchange is necessary for many of essential tasks associated with swidden agriculture, normative reasoning is necessary to prevent over-harvesting, and reciprocity is necessary to prevent excessive sanctioning.

So far, we treated μ as a group parameter that applies to all agents in the group. However, within-group behavioural heterogeneity in real groups can significantly affect evolutionary dynamics [62–67]. To test the robustness of our main result on the key role of the balance between reciprocity and normative reasoning in the evolution of sustainable swidden agriculture, we introduce heterogeneity in the parameter μ, so that each individual $\mu_{i}$ is drawn randomly and independently from a normal distribution with mean $\bar{\mu }$ and s.d. $\sigma_{\mu }$. As shown in electronic supplementary material, figure S3, a relatively small variation in the parameter μ has no effect on the model dynamics. However, a significant variation in μ can undermine sustainability and lead to complete deforestation. Overall, a higher importance of normative reasoning is required to maintain sustainable swidden agriculture in a more heterogeneous group.

Finally, we examine the joint effects of group size N and the relative importance of normative reasoning μ on the model dynamics (figure 4b). We find that (i) deforestation is typically observed when N is relatively large and μ is small; (ii) a low-intensity swidden regime evolves if N is relatively small and μ is large; and (iii) a sustainable high-intensity swidden regime is typically observed at the transition between the two other regimes. The results indicate that the sustainable swidden regime is robust to changes in group size and supports our previous finding that reciprocity and sanctioning must be balanced in shaping helping behaviour to produce sustainable and intensive swidden dynamics, with this balance shifting towards greater importance of normative reasoning as group size increases. Indeed, in small groups, the ability of agents to over-harvest is explicitly limited by group size because the size of the labour exchange network is small. However, increasing N creates more opportunities for over-harvesting, so tighter sanctioning norms must evolve to promote sustainable swidden agriculture. Therefore, helping behaviour should be driven more by normative reasoning.

### 3.3. Effects of material factors

Here we examine how the cost parameters $c_{0}$ and c associated with helping behaviour shape the evolution of swidden agriculture. The results are shown in electronic supplementary material, figure S4. The main takeaway is that the evolution of sustainable high-intensity swidden agriculture is observed at intermediate values of the total cost parameter $\left(c+c_{0}\right)$. Indeed, with relatively small costs of helping, agents are motivated to increase their sanctioning thresholds y to expand their labour exchange network and receive higher pay-offs, which in turn results in rapid deforestation. In contrast, relatively high costs of helping significantly suppress helping behaviour leading to the evolution of severe sanctioning and low-intensity swidden.

We also test the joint effects of the cost parameter c and the relative importance of normative reasoning μ. As shown in electronic supplementary material, figure S5, the sustainable swidden regime is robust to changes in the structure of material pay-offs (e.g. costs), but we predict helping behaviour to be more driven by reciprocity as costs increase. The result is intuitive since both costs and the relative importance of normative reasoning are factors controlling the evolution of sanctioning thresholds $y_{i}$, which should be sufficiently high to promote intensive swidden agriculture and at the same time sufficiently low to maintain sustainability. Therefore, increasing costs create material selective pressures suppressing the sanctioning thresholds $y_{i}$, which should be compensated by social selective pressures promoting an increase in the thresholds that is caused by more reciprocal relations.

### 3.4. Effects of environmental factors

**Figure 5.**
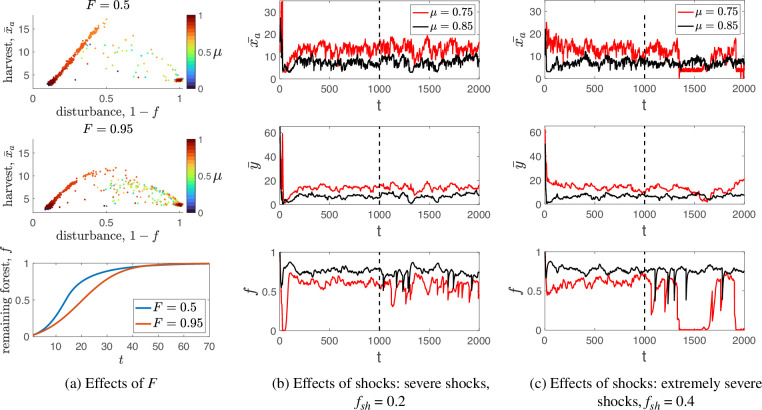
The effects of the environment on swidden dynamics. (a) Top and middle panels: the effects of parameter F on equilibrium levels of harvest and disturbance (1−f) (the effects on the amount of new biomass **$\Delta_{f}^{+}$** are shown in electronic supplementary material, figure S13). Results are shown for F=0.5 and F=0.95. Each point is the average of 100 runs for each value of μ∈{0,0.05,..,1}. The value of each run is an average of the final 250 rounds out of 1000 total rounds. Other parameters are at default values. Bottom panel: mean-field approximation (electronic supplementary material, section S2.2) for the reforestation curves in the absence of anthropogenic impact. (b-c) The effects of external shocks on swidden dynamics in terms of the average harvest ${\bar{x}}_{a}$, the sanctioning norm $\bar{y}$ and the remaining fraction of forest f. Shown are examples of the model dynamics for different values of shock severity $f_{sh}$ and the relative importance of normative reasoning μ. Shocks occur after t>T/2 (i.e. to the right to the vertical dashed lines). Parameters: $p_{sh}=0.01$. Other parameters are at default values.

Here we report how the interplay of environmental and social factors shapes the resulting swidden dynamics. First, we test the robustness of our results to changes in the environmental parameter F. We compare the model dynamics for F=0.5 and F=0.95, which represent different assumptions about the level of local forest density at which forest regeneration is most probable for a disturbed patch. As shown in figure 5a and electronic supplementary material, figure S13 and section S4.2, the main takeaway is that sustainable high-intensity swidden agriculture can evolve in various ecological settings but is more efficient in terms of harvests (figure 5a) and new biomass (electronic supplementary material, figure S13) at values of μ that lead to an intermediate level of forest disturbance. When F=0.5, an intermediate level of forest disturbance creates better local conditions for forest regeneration than when F=0.95, leading to higher harvests and new biomass. Moreover, for a fixed group size N and cost parameters c and $c_{0}$, a higher importance of normative reasoning μ is required to maintain a sustainable swidden regime with increasing parameter F. The result is intuitive: agents should be more constrained in their clearing requests in a forest ecosystem exhibiting less ability to recover from severe disturbances, so a higher importance of normative reasoning μ is required.

Second, we test the robustness of the sustainable high-intensity swidden regime to external environmental shocks. To model these shocks, we assume that each round, a shock occurs with a small probability $p_{s}$. This implies $f_{sh}L^{2}$ forest cells turn into empty cells, where $f_{sh}\in [0,1]$ captures the severity of the shocks, and if the remaining number of forest cells is less than $f_{sh}L^{2}$, all cells of the grid become empty. Figure 5b,c, electronic supplementary material, figure S6 (showing the results for F=0.5) and electronic supplementary material, figure S12 (showing the results for F=0.95) indicate that the sustainable high-intensity swidden regime demonstrates considerable resilience to increasing shock severity as long as the shocks are not extremely severe. In harsh environments characterized by extremely severe shocks, the sustainable high-intensity swidden regime can also be maintained, but the importance of normative reasoning μ must increase to ensure the evolution of tighter sanctioning norms in response to shocks.

So far, for the sake of parsimony, we followed the physics-based approach [52,53] to model the revegetation process by assuming that each empty cell turns into a forest with probability $p_{k}$ depending on the fraction of forest cells in the local neighbourhood. However, real reforestation processes are much more complex and typically involve time lags for vegetation recovery, which can capture the growth period necessary for a seed to become a tree, as well as vegetation succession. Here, to reflect this, we install a time delay mechanism by assuming that each empty cell transitions to a forest cell with probability $p_{k}$, but this only happens after the time lag l. We then examine the robustness of the model results to increasing the time lag l.

**Figure 6.**
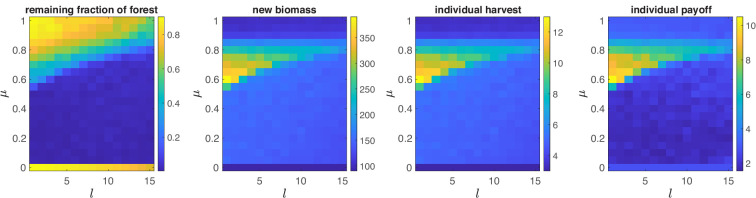
Joint effects of the relative importance of sanctioning μ and the time lag for revegetation l on: the remaining fraction of forest f, the new biomass $\Delta_{f}^{+}$, the group harvest $x_{a}$ and the group payoff π. Shown are the averages based on 100 runs each of 1000 rounds for each parameter combination. Results in each run are averages based on the last 250 rounds. N=30, other parameters are at default values.

As shown in figure 6, our main results are robust to increasing the time lag l. However, a higher relative importance of normative reasoning μ is required to maintain a sustainable high-intensity swidden regime with increasing parameter l. The result is intuitive, as increasing the time lag makes the revegetation process more difficult, so a more severe sanctioning should evolve to prevent overharvesting, which in turn requires a higher importance of normative reasoning μ. Intuitively, increasing l results in lower pay-offs obtained by the agents in the high-intensity swidden regime. Moreover, a high-intensity swidden regime can be maintained by reducing the group size N (e.g. through the exodus of some agents) to cope with significant increases in the time lag l. Finally, it should be pointed out that in reality, farmers have additional mechanisms to cope with long revegetation lags. For instance, they may cultivate fields for extended periods before allowing them to regenerate or by placing fields in riparian areas where annual flooding rejuvenates soils. Such adaptive strategies likely contribute to the robustness of the high-intensity swidden regime.

## 4. Discussion

Many assume that swidden agriculture typically results in deforestation because it is a common property system that is characterized by a lack of centralized regulation [68]. However, the ubiquity and persistence of swidden throughout human history suggests that swidden may be more sustainable than previously understood. Downey *et al*. [25] proposed that swidden agriculture is best viewed as a complex adaptive system: in the absence of top-down environmental managers or institutions, individual clearing decisions by farmers manifest emergent properties at the landscape scale related to the characteristics and overall state of the forest. Given this proposition, what are the social and ecological mechanisms or factors that could be responsible for maintaining the sustainability of swidden agriculture? The answer lies in the very nature of swidden cultivation: swidden farming involves multiple labour-intensive tasks that cannot be effectively performed individually but only within labour exchange groups, so that farmers depend on receiving help from others. Helping behaviour in many small-scale swidden communities tends to be driven by labour reciprocity but also by normative reasoning, that is to say, the process by which farmers decide whether to provide help in response to a specific clearing request depending on its perceived acceptability [22,30]. For example, in Maya villages in Southern Belize, farmers tend to withhold help (i.e. sanction) those who want to clear excessively large fields and tend to provide help to those who want to clear relatively small fields [27]. The results of behavioural experiments in these villages demonstrate that such normative reasoning leads to the emergence of a sanctioning norm that might be an important determinant of sustainable swidden agriculture. Here, we attempt to understand how clearing requests, perceptions of acceptability, sanctioning and forest ecology coevolve. Our model operationalizes a simple theory for how the social factors that govern labour exchange networks can also determine the emergent sustainable and unsustainable regimes of community forests. In doing this, we shed light on the role of labour reciprocity in this process and how socio-behavioural factors interact with demographic, material and environmental factors to shape the sustainability of this SES. Finally, we have mapped the full parameter space of possible outcomes related to labour reciprocity, normative reasoning and several simple ecological models of forest growth, under the assumptions of the model.

Our parsimonious mathematical model of swidden agriculture is built using a minimalist evolutionary approach. It includes complex decision-making mechanisms reflecting the bounded rationality of agents, and nuanced social dynamics associated with reciprocity and normative reasoning that are derived from ethnographic observations of swidden labour exchange. Three regimes are observed in our simulation results: a low-intensity swidden regime, a deforestation regime and a sustainable high-intensity swidden regime that maximizes both forest biomass productivity and harvest returns. Deforestation occurs when no significant sanctioning evolves to prevent environmental degradation. In the low-intensity swidden regime, an extremely severe sanctioning norm is observed, which breaks down labour exchange connections leading to small-scale individual swidden farming. As a result, the outcome is environmentally sustainable, but it is economically inefficient and would be unlikely to meet metabolic needs. In the sustainable high-intensity swidden regime, moderate sanctioning evolves, which prevents over-harvesting and encourages labour reciprocity that is essential to swidden agriculture in small-scale societies. This regime is theoretically sustainable for the forest and efficient for society, and we have identified the factors that contribute to the emergence of this sustainable and intensive swidden regime. The key finding is that the balance between normative reasoning and reciprocity in helping behaviour is crucial for the evolution of sustainable and intensive swidden agriculture: normative reasoning is needed to prevent over-harvesting, while reciprocity is necessary to prevent excessive sanctioning. Nevertheless, the system does not have to remain in balance over the short term—instead, it is important that agents have the ability to adjust their perceptions of allowable land use and clearing requests in order for the system to remain resilient to shocks and to recover from disturbances.

Importantly, our results also indicate that the sustainable high-intensity swidden regime exhibits considerable robustness to changes in demographic, material and environmental factors. Specifically, it can be robust to changes in group size, but agents in larger groups should condition their helping behaviour more by normative reasoning and less by reciprocity, so that tighter sanctioning norms can evolve. We also show that excessively small and excessively large groups are both inefficient swidden cultivators. In the former case, agents cannot harvest enough due to lack of sufficient labour partners, and in the latter case forests can become degraded. We find some evidence supporting our results in the ethnographic literature on Q’eqchi’ Maya villages in Belize showing considerable variance in village size with the highest reciprocity rate observed in the smallest village [22]. The results also demonstrate considerable flexibility of human behaviour to changes in the group size and composition. Indeed, a documented exodus of almost half the population of a village in 2008 and subsequent re-population by recruited families from other villages led to increased reciprocity rate, but not to settlement collapse [22].

We demonstrate that sustainable high-intensity swidden agriculture can evolve in a variety of ecological settings, but that both harvest and biomass productivity are most efficient when the balance between normative reasoning and reciprocity leads to an intermediate level of forest disturbance [69]. This is consistent with previous calls highlighting the importance of landscape disturbance for understanding how swidden agriculture is integrated into ecological dynamics [26,70], as well as remote sensing analysis which indicates that tree canopy diversity in Maya community forests is highest at intermediate levels of swidden disturbance [25]. As far as we are aware, ours is the first theoretical study to implement a forest growth model which shows that a combination of reciprocity and normative reasoning can lead to intermediate levels of forest disturbance that enhance both agricultural and ecological productivities. In addition, our results show that the sustainable high-intensity swidden regime exhibits considerable resilience to increasing severity of environmental shocks, negatively affecting the forest as long as the shocks are not extremely severe. With extremely severe shocks, a sustainable swidden regime can also be reached, but the likelihood of the settlement collapse is much higher. Alternatively, the relative importance of normative reasoning μ needs to be increased to ensure the evolution of tighter sanctioning norms to cope with the extremely severe shocks. This is well in line with ethnographic observations that although forest-damaging hurricanes are quite common in Belize [71,72], many Maya communities are able to cope with these shocks. Moreover, our theoretical findings support recent work [73] suggesting that Maya communities should be able to adapt and recover from the outbreak of climate-change driven wildfires that affected Belize in 2024 [74] if they are supported in a culturally appropriate manner.

**Figure 7.**
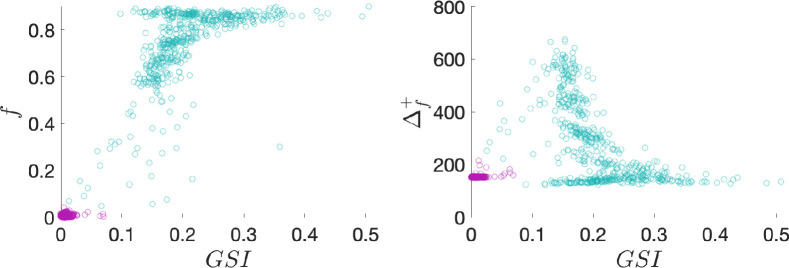
The effects of the graduated sanctioning index (GSI) on the remaining fraction of forest f (left panel) and the new biomass $\Delta_{f}^{+}$ (right panel). Shown are averages in different runs (points). Violet points correspond to unsustainable runs (i.e. those with f≤0.05), while green points correspond to sustainable runs (i.e. those with f>0.05). The relative importance of sanctioning μ∈{0.5,...,1} (simulations converge at a deforestation state when μ<0.5). Other parameters are at default values. For each value of μ, we performed 100 independent simulation runs and in each run, the simulation consists of 1000 rounds. Results in each run are averages based on the last 250 rounds.

In addition, we compare our modelling results with the previous experimental findings in Maya swidden communities [27], demonstrating that sanctioning through swidden helping networks leads to more sustainable ecosystem outcomes. To show this in the model, we compute the graduated sanctioning index (GSI), a standardized measurement of the difference between each player’s clearing decision and the help they received in each round [27]. As shown in figure 7 (left panel), higher GSI values are associated with greater forest retention, aligning with the experimental results reported in fig 6 in [27]. Moreover, the model predicts that ecosystem productivity, measured in terms of the new biomass, follows a hump-shaped relationship with the GSI (figure 7, right panel). This suggests that intermediate levels of sanctioning yield the most beneficial outcomes for both the forest and the people. Furthermore, the model results reveal significant variability in ecological outcomes. This is a consequence of the fact that the model exhibits bi-stability around the high-intensity swidden–deforestation bifurcation threshold. As a result, depending on the initial conditions, various ecological trajectories can emerge. This may explain the substantial variability observed among different groups in the experimental Milpa game [27].

In §1, we noted that our model is most comparable with an archaeological agent-based model of swidden agriculture developed by Michael Barton. The main question his model explores is whether swidden farming in an environment of rapidly exhausted soils by a small number of households can produce an evenly distributed spatial pattern of sites [21, p. 213]. The model includes an adaptive version similar to our strategy revision protocol, which uses a genetic algorithm to initialize households with randomized parameters, a fitness function and parameter inheritance, and a cluster analysis to determine subsets of the parameter space that result in more successful/adaptive farming strategies. In other words, the adaptive version of the Barton model is a type of genetic model where daughter villages copy their parents’ alleles related to framing strategies and ‘bud off’ from successful parent villages. Another interesting similarity between our modelling results and Barton’s is the importance of social norms for achieving sustainability. Barton’s analysis finds that norms regarding household land tenure decisions can create emergent patterns that in this case reduce undesirable population fluctuations. In our model, labour exchange and normative reasoning are key drivers. However, the overarching goal of Barton’s model is ultimately to provide insight into the social mechanisms related to swidden agriculture that could lead to the settlement patterns that are observed archaeologically. This differs from, but is ultimately complementary to, our stated goal, which is to understand how social dynamics relate to ecosystem dynamics *within* a swidden forest of a single community.

In closing, we note that while our model operationalizes complex dynamics related to social norms, swidden labour and forest ecology in a simple and mathematical way, individual decision-making in swidden communities—as indicated by previous ethnographic research—is a much more complex psychological and strategic process. Indeed, determining field sizes, desired harvest levels and managing one’s labour partnerships in the real world involve considering household composition, economic need, time allocation, limited information, assessment of opportunity costs and risk. For example, a household survey we conducted in 2018 indicated that social factors including kinship and church membership, and economic factors such as cattle ranching, have statistically significant effects on the structure of normative labour exchange networks [30]. Adding kinship networks to the model would increase the probability that two agents who were kin would cooperate with each other, irrespective of their individual sanctioning thresholds, increase average labour reciprocity rates and therefore increase the average likelihood of the deforestation regime. However, this tendency can theoretically be balanced out by shifting the relative importance of normative reasoning, so the increased rate of labour reciprocity between kin could, in principle at least, be balanced out by higher levels of sanctioning. We also note that it is the purpose of modelling to simplify this reality to better understand the underlying social and ecological dynamics, which we do by focusing on a limited set of factors, labour reciprocity and normative reasoning.

Myopic optimization and pay-off-based imitation are parsimonious techniques to model processes of strategic behaviour, social learning and adaptation that are significantly more complicated in the real world. Nevertheless, in using these approaches, we illustrate one way it can occur, and in doing so, we hope to better understand the underlying dynamics of swidden agriculture and to provide a plausible demonstration of self-organization in swidden agricultural systems. We also focus attention on modelling social dynamics related to labour reciprocity and normative reasoning, and model forest dynamics in a very minimalist way. This decision was guided by our examination of the swidden modelling literature, which we found to be missing models explaining how social regulation of environmental processes can occur. Modelling a wider range of forest succession stages and the effects of ecological dynamics such as seed dispersal on harvest levels, ecosystem diversity and productivity would make the model more realistic and potentially useful for understanding real environments [25], although increasing significantly its complexity. Finally, our current model focuses exclusively on individual decisions about field sizes; however, field location and land tenure practices [21] are also important determinants of swidden cultivation. Overall, we believe that incorporating individual decisions on the location of new clearings along with the spatial dependencies of costs, harvest and forest regeneration will also help explain the real patterns of swidden mosaics, for example, as they are observed in remote sensing data [25]. We leave these extensions of the model for future work.

## 5. Conclusion

Swidden agriculture is one of the most widely studied examples of a coupled human and natural system, yet the scarcity of formal models incorporating both its social and environmental dynamics is surprising and consequential. Previous ethnographic fieldwork with Maya communities in Belize has identified key social dynamics, such as reciprocal swidden labour exchange networks, and ecological dynamics, like forest disturbance that, when combined with concepts from CAS theory, capture fundamental aspects of swidden that we believe are relevant to many societies worldwide, in the past, present and future. In this study, we have distilled a parsimonious model of village-scale social norms related to agricultural labour that interacts with forest disturbance ecology, that is capable of producing a wide range of emergent sustainable and unsustainable outcomes. Our simulation results suggest that the model is resilient across diverse ecosystem types, robust to various perturbations and that it generates intuitive results like under-utilization and deforestation, as well as counter-intuitive outcomes such as enhanced harvests and biomass productivity (e.g. carbon sequestration). These qualitative characteristics are expected in swidden systems, which is a persistent and widespread subsistence strategy throughout human history. We believe our theoretical model may have significant implications for understanding the role that small-scale and Indigenous swidden societies can play in global efforts against climate change. Importantly, our model does not rely on top-down ecosystem management or formal institutions to achieve sustainability. This study focuses on developing and documenting a theoretical model that can be reproduced, tested against empirical observations and adapted for further scientific research and policy discussions. Overall, we suggest that simple models of swidden agriculture, faithful to observed social and ecological dynamics and able to account for real-world patterns, are essential for advancing our understanding of swidden agriculture and informing policy development in the modern era of climate change.

## Data Availability

The simulations were performed in MATLAB (v. R2024a). The code is provided in electronic supplementary material, section S5 [75].

## Footnotes

^1^
The maximum number of cells each player was allowed to clear each round of the game was five.

^2^
Note that we consider a Moore neighbourhood, meaning that each interior cell of the grid has eight neighbours.

^3^
We postulate $q_{i,j}=1$ if i=j.

^4^
Note this effect only holds if costs of helping are sufficiently high.

^5^
Note that in sustainable outcomes, f is typically greater than 0.5. Some analytical insights explaining this phenomenon can be found in electronic supplementary material, section S2.2.
